# Determinants of trajectories of fatigability and mobility among older medical patients during and after hospitalization; an explorative study

**DOI:** 10.1186/s12877-021-02714-9

**Published:** 2022-01-03

**Authors:** Marlies Feenstra, Barbara C. van Munster, Nynke Smidt, Sophia E. de Rooij

**Affiliations:** 1 Department of Internal Medicine and Geriatrics, University of Groningen, University Medical Center Groningen, P.O. Box 30001, 9700 RB Groningen, The Netherlands; 2 Department of Geriatrics, Gelre Hospitals, Apeldoorn, The Netherlands; 3 Department of Epidemiology, University of Groningen, University Medical Center Groningen, Groningen, The Netherlands

**Keywords:** Older people, Fatigue, Physical activity, Longitudinal Studies, Geriatrics

## Abstract

**Background:**

Fatigability is an important marker of functional decline in community dwelling older people, yet its relationship with functional decline after hospitalization is unclear. The objectives of this study were to identify trajectories of fatigability and mobility over time and to examine the association between demographic and clinical characteristics and these trajectories in medical patients aged 70 years and older admitted to a Dutch tertiary care teaching hospital.

**Methods:**

In this prospective cohort study with baseline (in-hospital), discharge, three-, and six-months post discharge follow-up measurements, fatigability was assessed by the physical subscale of the Pittsburgh Fatigability Scale (PFS). Mobility was assessed by the De Morton Mobility Index (DEMMI). Group-based trajectory modeling was used to identify joint trajectories of fatigability and mobility. Covariates included demographic (age, sex, living situation, education) and clinical characteristics (functional status, frailty status, depression, comorbidity, length of hospital stay).

**Results:**

Among 44 patients, three distinct fatigability trajectories and two mobility trajectories were identified over the course from hospital admission up to six months after discharge. Subsequently, three joint trajectories were identified, including low fatigability and high mobility (11%), improving fatigability and high mobility (52%), and high fatigability and low mobility (36%). Controlling for baseline functional status, patients with a lower comorbidity score (OR: 0.27, 95%CI 0.10; 0.74) and higher frailty status (OR: 1.36, 95%CI: 1.07; 1.74) were more likely to be a member of the high fatigability and low mobility trajectories.

**Conclusions:**

From hospital admission up to six months after discharge, three distinct trajectories of fatigability and mobility were identified among older medical patients. Our results should be interpreted with caution due to the small sample size, but may inspire other researchers to determine the value of fatigability assessment in identifying older medical patients at risk for developing mobility problems.

**Supplementary Information:**

The online version contains supplementary material available at 10.1186/s12877-021-02714-9.

## Background

Fatigue is the most prevalent symptom among older hospitalized patients, with a prevalence rate of around 70% [1, 2]. After hospital discharge, many older people still experienced fatigue, often leading to disruption of activities of daily living (ADL) [3]. Hence, it can be assumed that older hospitalized patients experience high prevalence of fatigability as well, which is defined as perceptions of fatigue while performing activities of a certain intensity and duration [4]. Over the past decade, fatigability has emerged as an important marker of functional decline in community dwelling older people without disabilities [5–7], yet its relationship with functional decline after hospitalization is unclear.

Functional decline is a serious consequence of hospitalization among older patients, with a prevalence rate of around 30% at the moment of discharge [8]. Recovery rates of functional decline varied from 30% to 70% at three months after discharge from the hospital [9–12]. Low mobility during hospital stay is an important predictor of adverse outcomes after hospitalization such as functional decline, re-hospitalization, institutionalization, and death [13, 14]. In addition, loss of mobility and exhaustion or fatigue are considered as early manifestations of frailty, contributing to functional decline and dependence in older people [15, 16]. The association between mobility and fatigability among hospitalized older patients has not been directly investigated yet, but it may provide interesting information to design targeting interventions to prevent adverse outcomes after hospitalization of older patients.

Therefore, this study aimed to investigate whether trajectories of fatigability are associated with trajectories of mobility from hospital admission up to six months post-discharge in medical patients aged 70 years and older. A second aim is to investigate the determinants of these combined trajectories.

## Methods

### Participants

Patients admitted to medical wards of a tertiary care teaching hospital in the Netherlands were recruited for participation within the first four days of hospital admission. Patients younger than 70 years, those who did not speak Dutch, those who were cognitively impaired due to dementia or delirium, and those who did not provide informed consent were excluded from participation.

### Procedure

Every weekday between April 2018 and July 2019, all consecutive patients admitted to the hospital were screened for eligibility by trained research staff. The research staff introduced the study to eligible patients and handed them written information about the study procedure. The next day patients were visited again to ask for their decision to participate. All patients signed informed consent before enrollment. The study was approved by the local research ethical committee (registration number: 2017.667).

### Data collection

Baseline assessment was conducted within the first four days of hospital admission by trained research staff. Data collection consisted of comprehensive questionnaire and physical assessments. Follow-up assessments were conducted within 24 hours before the expected discharge time, based on daily consultation with the chief nurse of the wards involved (only when discharge was at least three days after baseline assessment), three months after discharge (home visit), and six months after discharge (home visit). An overview of the moments and type of assessments is provided in ***Supplementary Table*** ***S1***.

- Demographic variables included age (in years), sex (male, female), living situation (living alone, co-habiting) and educational level (≤12 years; >12 years of education).
- Clinical variables included: 1. Frailty status measured by the Frailty Index including 34 health-related items such as psychosocial, cognitive, and general functioning, and geriatric symptoms [17]. Item scores were rescaled into values between 0 and 1, with higher scores representing worse functioning. The total score represents the proportion of present items as a proportion of all items measured, resulting in a Frailty Index score ranging from 0 to 1. A detailed item description is presented in ***Supplementary Table*** ***S2***; 2. Pre-hospital functional status (re-call based) measured by the 15-item Katz ADL index score including basic and instrumental ADL [18]; 3. Depressive symptoms were screened by using the two questions ‘During the past month, have you often been bothered by feeling down, depressed or hopeless?’ and ‘During the past month, have you often been bothered by little interest or pleasure in doing things?’ [19]. If one or two of these screening questions were answered with ‘yes’, the 15-item GDS was subsequently assessed using a cut-off of five items for depression [20, 21]; 4. Chronic conditions were measured by the Charlson Comorbidity Index (CCI) [22]; and 5. Length of hospital stay was obtained from the hospital medical records after hospital discharge.
- Fatigability was measured by the Pittsburgh Fatigability Scale (PFS) [23]. The PFS was originally developed in the United States as a self-administered scale consisting of 10-items on which respondents indicated the level of perceived physical and mental fatigue that they expect to perceive immediately after performing the activity on a scale from 0 (no fatigue at all) to 5 (extreme fatigue). Activities include physical activities, household activities, sedentary activities, and social activities. Total physical and mental fatigability scores range from 0-50, with higher scores indicating higher fatigability. The PFS was recently translated into Dutch and validated in older hospitalized patients, showing good internal consistency (range α: 0.80 - 0.92), and test-retest reliability (ICC: 0.80-0.81) [24]. For this study, only the PFS physical score was used.
- Mobility was measured by the ‘De Morton Mobility Index’ (DEMMI) [25]. The DEMMI is a physical performance test designed in Australia for older medical patients consisting of 15 mobility tasks that should be performed by the patient while lying (3 items), sitting (3 items), standing (4 items) and moving (5 items) [25]. A trained research assistant rated for each item whether the patient was able to perform the activity without help, with minimal assistance, or not at all. The total score ranged from 0 (no mobility) to 100 (full mobility). The Dutch version of the DEMMI was successfully examined for inter-rater reliability (0.85) and construct validity with other physical performance tests (ρ ranging from 0.73 to 0.74) among older hospitalized patients [26].

All instruments used are licensed for non-commercial research purposes provided the original authors are referenced.

### Statistical analysis

First descriptive statistics including demographic and clinical characteristics of the total study sample were calculated. Next, group-based trajectory modeling (GBTM) was applied to identify trajectories of fatigability and mobility. Here, a stepwise approach was used [27]:

- Step 1, separate models: separate fatigability and mobility trajectories were plotted using a censored normal model with fixed cubic growth terms for both models. The number of trajectory groups was selected using Bayesian Information Criterion (BIC) [27]. Second, higher or lower order growth terms were added, and optimal trajectory shape was determined based on posterior diagnostic criteria such as 95% confidence intervals, odds of correct classification, and posterior probability of assignment. These posterior diagnostic criteria reflect the probability of a person belonging to the selected trajectory and are explained in detail elsewhere [28].
- Step 2, dual models: Next, the trajectories of fatigability and mobility obtained in the first step were simultaneously modeled. This so called dual trajectory model linked the trajectories of fatigability with the trajectories of mobility by estimating the conditional probabilities of membership in the trajectory groups of fatigability, given membership in one of the specific trajectory groups of mobility.
- Step 3, adding covariables: Finally multinomial logistic regression analyses were done to explore the associations between the identified dual trajectories and demographic and clinical co-variables. Baseline age, sex, educational level, living situation, functional status, frailty status, depression, and length of hospital stay were alternately (univariably) added as independent variable using the estimated dual trajectory groups as dependent outcome. Given the complex interactions between fatigability, immobilization, disability, frailty, and comorbidity [15, 16, 29], a multivariable model was built including these variables to explore potential confounding effects. For these variables, presence of multicollinearity was checked using Pearson’s correlations. Odds ratios and 95% confidence intervals of each co-variable per trajectory group were presented.

Subjects with missing data at all time points were excluded from the analysis. In the trajectory analysis, missing data for some time points were imputed using maximum likelihood estimation. Items of the PFS were imputed using item mean imputation when a. the question whether the activity had been done in the past month was present, and b. maximum three items of the PFS physical subscale were missing [30]. For all analyses Stata Statistical Software release 14 was used (StataCorp. 2015. College Station, Texas, USA) using the Traj plug-in [31].

## Results

### Characteristics of study participants

**Fig. 1:**
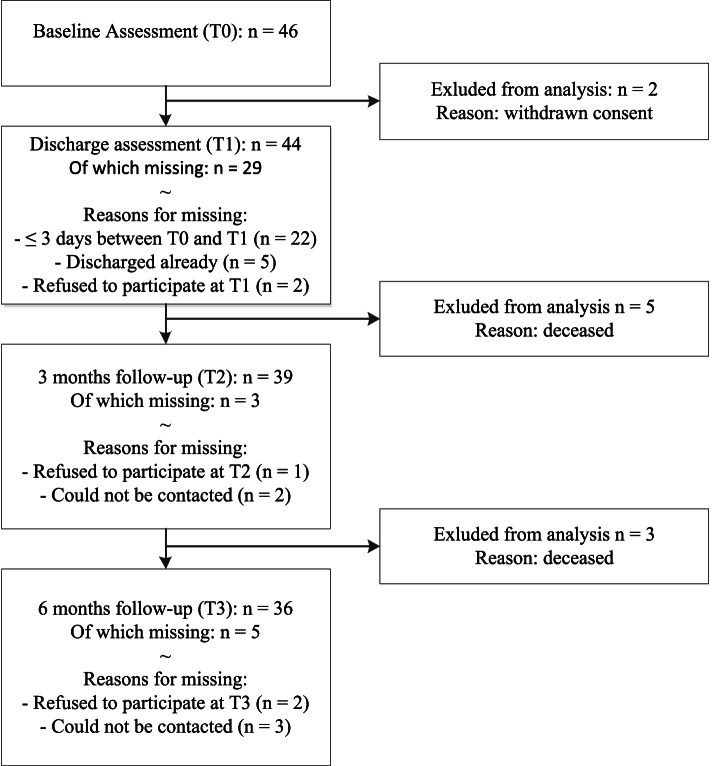
Flowchart of study participants

Of the 843 eligible hospitalized medical patients aged 70 years and older, 46 gave written consent to participate in our study. Two patients were excluded from the analyses due to withdrawn consent during baseline assessment, leaving 44 patients in the analytic sample. A flowchart of study participants is presented in ***Figure*** ***1***. Demographic and clinical characteristics of study participants are presented in Table 1. Among all participants, sex was equally distributed, median age was 75 years ranging from 70 to 88 years, and the median length of hospitalization was five days (IQR: 3; 8). The majority of the study population lived together (70%) and had one or more disabilities in performing ADL before hospital admission (60%). The minority of the study population had less than twelve years of education (42%) and had five or more depressive symptoms at baseline assessment (18%). The median comorbidity index score of the total group was 2 (IQR: 1; 3) and a detailed description comorbidity distribution is provided in ***Supplementary Table*** ***S3***. The majority of the patients (86%) were admitted for therapeutic intent, receiving curative treatment (***Supplementary Table*** ***S4***).

**Table 1:** Baseline characteristics of all participants (n=44) and according to the joint trajectories of fatigability and mobility

Baseline characteristic	Total	Trajectory group^*^		
		Low fatigability high mobility	Improving fatigability high mobility	High fatigability low mobility
**N (%)**	44 (100)	5 (11)	23 (52)	16 (36)
**Demographic characteristics**				
Age in years, median (IQR)	75 (73; 81)	75 (74; 81)	75 (71; 79)	79 (73; 83)
Female sex	22 (50)	2 (9)	10 (45)	10 (45)
<12 years of education	18 (42)	1 (6)	7 (39)	10 (56)
*Missing*	*1 (3)*			
Living alone	13 (30)	2 (15)	5 (38)	6 (46)
**Clinical characteristics**				
Frailty^†^, median (IQR)	0.26 (0.12; 0.34)	0.12 (0.04; 0.20)	0.15 (0.10; 0.32)	0.35 (0.29; 0.49)
≥1 disabilities	29 (66)	1 (3)	13 (45)	15 (52)
*Missing*	*1 (3)*			
≥1 disabilities basic ADL	18 (41)	0 (-)	8 (44)	10 (56)
≥1 disabilities iADL	26 (60)	1 (4)	12 (46)	13 (50)
*Missing*	*1 (3)*			
Depressive symptoms^‡^	8 (18)	1 (13)	2 (25)	5 (63)
Comorbidity^§^, median (IQR)	2 (1; 3)	3 (2; 5)	2 (1; 3)	2 (1; 3)
*Missing*	*2 (5)*			
LoS in days, median (IQR)	5 (3; 8)	4 (2; 5)	4 (3; 7)	7 (5; 9)
Fatigability^|^, mean (SD)	31 (9)	14 (3)	29 (7)	37 (5)
*Missing*	*9 (20)*			
Mobility^¶^, mean (SD)	65 (23)	76 (24)	78 (13)	44 (18)
*Missing*	*5 (11)*			

Results are presented as n (%) unless indicated otherwise. Percentages may not add up to 100 due to rounding. The number of missing values at baseline are presented, unless there were no missing values.

^*.^Trajectory groups were estimated by jointly modeling fatigability and mobility over four waves from hospital admission up to six months after discharge.

^†.^Frailty was assessed by the Frailty Index.

^‡.^Depressive symptoms were positive when the Geriatric Depression Score was ≥5.

^§.^Comorbidity was assessed by the Charlson Comorbidity Index.

^|.^ Fatigability was assessed by the physical subscale of the Pittsburgh Fatigability Scale.

^¶^Mobility was assessed by the De Morton Mobility Index.

Abbreviations: ADL, activities of daily living; IQR, interquartile range; LoS, length of stay.

Baseline characteristics of patients with missing data on the PFS physical subscale did not differ from patients with complete data (***Supplementary Table*** ***S5***), nor did we identify missing assessments of only the most vulnerable patients. Consequently, these types of missing data were assumed to be missing completely at random. Therefore the data of five patients (11%) with less than three missing items of the PFS physical subscale were imputed, and maximum likelihood estimation was applied in the trajectory analyses when one or more assessments were missing.

### Separate fatigability and mobility trajectory models

Among all evaluated trajectory models, a 3-group trajectory solution was selected as the optimal model for fatigability and a 2-group trajectory solution was selected as the optimal model for mobility. The posterior diagnostic criteria of the selected models were good, indicated by the margins of error <1%, the odds of correct classification >5, and the posterior probabilities of assignment ranging from 0.85 to 0.94 for the fatigability trajectories and from 0.95 to 0.96 for the mobility trajectories.

### Fatigability trajectories

Using repeated measures of four waves of the PFS physical subscale, three fatigability trajectories were identified: stable low fatigability (n: 5, 13%), improving fatigability (n: 19, 43%), and stable high fatigability (n: 20, 45%).

### Mobility trajectories

Using repeated measures of four waves of the DEMMI, two mobility trajectories were identified: stable low mobility (n: 15, 34.2%) and stable high mobility (n: 29, 65.8%).

### Dual trajectory model of fatigability and mobility

**Fig. 2:**
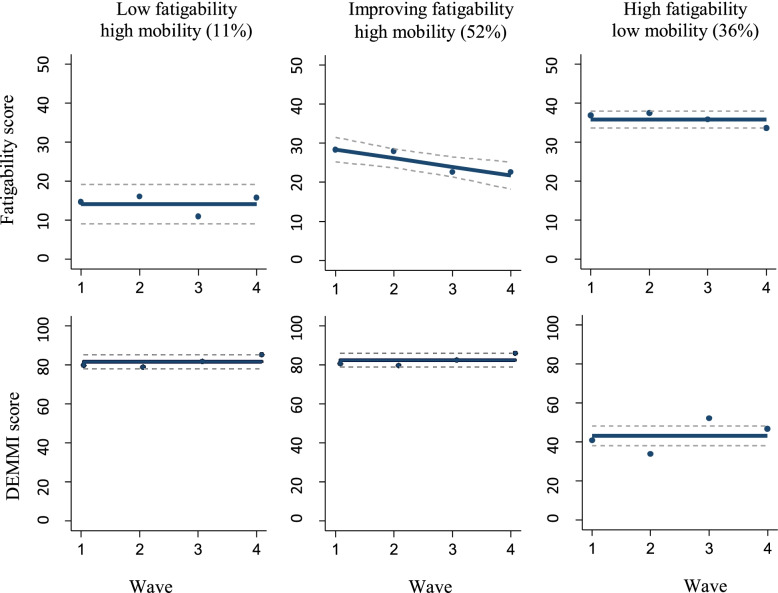
Joint trajectories of fatigability and mobility. Fatigability was assessed using the Pittsburg Fatigability Score physical subscale (PFS, score range: 0 - 50). Mobility was assessed using the De Morton Mobility Index (DEMMI, score range 0 - 100). Higher scores represent higher fatigability and mobility. Dots represent mean predicted PFS and DEMMI scores. Dashed lines represent 95% confidence intervals of the mean predicted PFS and DEMMI scores

Table 2 represents the posterior probabilities of each fatigability trajectory membership given the mobility trajectory (A) and the posterior probabilities of each mobility trajectory given the fatigability trajectories (B). Here it is shown that the fatigability trajectory largely determined the mobility trajectory someone is allocated to: 86% of the patients who were assigned to the stable low fatigability trajectory were assigned to the high mobility trajectory as well (p<0.001); all of the patients (100%) assigned to the improving fatigability trajectory were assigned to the high mobility trajectory too (p<0.001); and, 74% of the patients who were assigned to the high fatigability trajectory were assigned to the low mobility trajectory (p<0.001). Figure 2 presents these three trajectory combinations resulted from jointly modeling the fatigability and mobility trajectories: 1. Low fatigability and high mobility (11%); 2. Improving fatigability and high mobility (52%), and 3. High fatigability and low mobility (36%). Baseline characteristics of the study participants by these trajectory combinations are presented in Table 1. Table 2**-C** presents the posterior probabilities of assignment to each of these dual trajectory groups.

**Table 2:** Comparison of posterior probability of assignment for the single mobility model, the single fatigability model and the trajectory model that jointly modelled mobility and fatigability

A. Fatigability given mobility trajectory: posterior probability of assignment				
		Fatigability trajectory groups		
		Stable low (n=5)	Improving (n=19)	Stable high (n=20)
Mobility trajectory groups				
Low (n=15)		0.05	0.00	0.95
High (n=29)		0.16	0.66	0.17
B. Mobility given fatigability trajectory: posterior probability of assignment				
		Mobility trajectory groups		
		Low (n=15)	High (n=29)	
Fatigability trajectory groups				
Stable low (n=5)		0.15	0.86	
Improving (n=19)		0.00	1.00	
Stable high (n=20)		0.74	0.26	
C. Joint fatigability mobility model: posterior probability of assignment				
Group Allocation	N (%)	Low fatigability high mobility	Improving fatigability high mobility	High fatigability low mobility
Low fatigability high mobility	5 (11)	0.79	0.03	<0.001
Improving fatigability high mobility	23(52)	0.06	0.79	0.01
High fatigability low mobility	16 (36)	<0.001	0.01	0.86

### Associations between dual trajectory and covariables

The associations between baseline demographic and clinical characteristics and dual trajectory group membership probabilities using the most favorable trajectory group as the reference group are presented in Table 3. The results of the univariable multinomial logistic regression analysis showed that presence of ADL disabilities before hospital admission (OR: 60.00, 95%CI: 3.04; 1185.03) and a higher frailty index (OR: 1.26, 95% CI: 1.08; 1.46) more often lead to membership of the high fatigability and low mobility trajectory group. The results of the multivariable multinomial logistic regression analysis showed that, after adjustment for baseline ADL disabilities, patient with a lower comorbidity index score (OR per point increase: 0.27, 95%CI 0.10; 0.74) and higher frailty status (OR per 0.01 point increase: 1.36, 95%CI: 1.07; 1.74) more often belonged to the high fatigability and low mobility trajectory group (Table 3). For completeness, the results of all univariable analyses using the improving fatigability, high mobility as the reference group, the univariable regression estimates for the separate mobility and fatigability trajectories, and the correlation matrix of all covariables are presented in Supplementary Tables S6, S7, S8, and S9, respectively.

**Table 3:** Regression estimates (odds ratio’s and 95% confidence intervals) of the associations between the dual fatigability and mobility trajectories^*^ and demographic and clinical characteristics

Characteristic	Low fatigability high mobility	Improving fatigability high mobility	High fatigability low mobility
**Univariable analysis**			
**Demographic characteristics**			
Age, per year	Ref.	0.94 (0.79; 1.13)	1.03 (0.86; 1.23)
Female sex	Ref.	0.87 (0.12; 6.21)	0.00 (0.05; 3.12)
Male sex	Ref.	Ref.	Ref.
<12 years of education	Ref.	0.57 (0.05; 6.08)	0.12 (0.01; 1.43)
≥12 years of education	Ref.	Ref.	Ref.
Living alone	Ref.	0.42 (0.05; 3.22)	0.90 (0.12; 7.03)
Living together	Ref.	Ref.	Ref.
**Clinical characteristics**			
Frailty^†^, per 0.01 point	Ref.	1.08 (0.96; 1.21)	1.26 (1.08; 1.46)
No baseline disabilities	Ref.	Ref.	Ref.
≥1 baseline disabilities	Ref.	5.78 (0.55; 60.60)	60.00 (3.04; 1185)
No depressive symptoms	Ref.	Ref.	Ref.
Depressive symptoms^‡^	Ref.	0.38 (0.03; 5.27)	1.82 (0.16; 20.71)
Comorbidity^§^, per point	Ref.	0.74 (0.47; 1.19)	0.77 (0.48; 1.26)
Length of stay, per day	Ref.	1.11 (0.78; 1.59)	1.25 (0.87; 1.79)
**Multivariable analysis**			
Frailty^†^, per 0.01 point	Ref.	1.09 (0.91; 1.30)	1.36 (1.07; 1.74)
No baseline disabilities	Ref.	Ref.	Ref.
≥1 baseline disabilities	Ref.	5.04 (0.23; 109.14)	9.89 (0.14; 686.83)
Comorbidity^§^, per point	Ref.	0.56 (0.41; 1.12)	0.27 (0.10; 0.74)

^*^Trajectory groups were estimated by jointly modeling fatigability and mobility over four waves from hospital admission up to six months after discharge. The low fatigability high mobility trajectory was used as the reference category (Ref.)

^†.^Frailty was assessed using the Frailty Index

^‡.^Depressive symptoms were positive when the Geriatric Depression Score was ≥5

^§^Comorbidity was assessed using the Charlson Comorbidity Index

## Discussion

The current study identified three distinct fatigability trajectories and two mobility trajectories from hospital admission up to six months after discharge. Combining these two models revealed three joint trajectory groups of fatigability and mobility. The smallest group (11%) had the most favorable outcome with low fatigability and high mobility; the majority of the patients (52%) were allocated to the improving fatigability and high mobility trajectories; and 36% of the patients were allocated to the least favorable trajectories of high fatigability and low mobility. Controlling for baseline ADL disability, patients with a lower comorbidity index score and higher frailty index scores were more likely to be a member of the least favorable joint trajectory group: the high fatigability and low mobility trajectories.

Univariable analyses identified baseline ADL disability and frailty as determinants of the least favorable joint trajectory group. However, the multivariable analysis identified a lower comorbidity index score as a determinant of the least favorable combined fatigability and mobility trajectory group as well. This finding was not surprising given the complex and cyclic interplay of various factors that contribute to the manifestation of frailty such as immobility and fatigue: comorbidity and disability often co-occur together with frailty, but they are not conditional [15, 29]. In our study, patients with the highest probability of belonging to the most favorable trajectory group had more severe comorbidities (kidney disease, malignant tumor, metastatic tumor) that had a weighting factor of three or six on the total comorbidity index score. Lung disease and heart failure, on the other hand, were more common in people in the other two trajectory groups, which in themselves are less lethal than, for example, a metastatic tumor, but have a direct and profound impact on mobility and fatigability, which may explain the found associations between a lower baseline comorbidity index score and a higher likelihood of belonging to the high fatigability and low mobility trajectory group. Future studies should reveal whether using a more generic outcome that count the co-occurrence of multiple chronic conditions without weighting will yield the same results.

Patients with severe cognitive impairment, such as dementia or delirium, were excluded from participation, because the Pittsburgh Fatigability Scale was not yet validated in cognitive impaired populations. When these more vulnerable patient would have participated, more subgroups may have been identified within the sample, and the found differences between the best and worst identified subgroups may have been even larger. Nevertheless, the differences found between the mean fatigability score of the best and worst trajectories already exceeded the predefined smallest detectable change scores [24], indicating real subsamples. However, the number of trajectories is at odds with the number of patients within each trajectory [27], thus increasing the sample size is the main challenge for future research into trajectories of fatigability and mobility in older hospital patients.

Where previous research has identified fatigability to be a marker of mobility decline in community dwelling older adults [5, 6], our findings suggest that fatigability is associated with mobility levels of older medical patients as well. More specifically, patients with initial high, but improving fatigability levels over the six months following hospital discharge were more likely to have high mobility levels over the same period. By contrast, patients in the constant high fatigability trajectory were more likely to keep low mobility levels over time. Interestingly, a higher frailty index score appeared one of the determinants of this latter group. In the cycle of frailty, reduced strength and aerobic capacity, as a result of physiologic dysregulation due to aging processes in general or underlying disease, negatively affect physical performance outcomes such as walking speed and grip strength, which, in turn, affects functional capacity and the ability to remain physically active [32]. In this cascade of accumulating physiological dysregulations, exhaustion is, next to weight loss, a critical symptom of frailty [16]. In this light, the failure to restore fatigability, operationalized as the perceived fatigue during physical activities, may be one of the manifestations of frailty in hospitalized older patients as well, rather than that frailty is just a determinant of the least favorable joint trajectory. However, our results should be interpreted with caution, due to the small study sample, and larger studies are strongly recommended to determine the value of fatigability assessment in identifying older medical patients at risk for developing mobility problems and frailty.

Strengths of this study included repeated measures of comprehensive fatigability and mobility assessments that were collected by face-to-face contact, allowing us to investigate the development and interactions of these constructs longitudinally and secured high reliability of the collected data. There are also a number of limitations to address. First, despite this study was initially designed to investigate associations between fatigue, mobility and clinical patient characteristics, the low inclusion rates have led to underpowered results, as demonstrated by the relatively wide confidence intervals, indicating unreliable estimates which limits to draw firm conclusions [33]. Second, the small sample size forced us to include only a limited number of predictors in the multivariable model, excluding serious candidate predictors and control variables, such as patient characteristics, reason for admission and the principle diagnosis. Future studies should ensure a detailed description of the reason for admission and the main diagnosis to include them as control variables when modeling trajectories of mobility and fatigability. Third, to our knowledge, this is the first study that identified trajectories of fatigability over time, limiting mutual comparisons with previous studies investigating trajectories of fatigability over time. Fourth, the follow-up period involved only six months after hospital discharge, while there are indications that recovery of hospital admission among older adults can take up to two years [10].

To optimize the benefits and costs of targeted interventions to improve health outcomes in older people, it is important to identify people who need it most. Our results show that in-hospital fatigability assessment, supplemented with the DEMMI, could offer a screening tool that can be performed at the bedside, to identify patients that are prone to worse functional outcomes after hospitalization. Based on the trajectories identified in this study, interventions aiming at improving fatigability may be most effective when directed towards patients with high fatigability but a high mobility level during the first days of hospitalization. However, due to the small sample size in the current study, larger studies with preferably longer follow-up periods are needed to determine the value of fatigability assessment in identifying older medical patients at risk for developing mobility problems and who might benefit from tailored interventions.

## Conclusions

In this exploratory study in older medical patients, three major distinct trajectories of fatigability and mobility from hospital admission up to six months after discharge have been identified. Controlling for baseline disabilities in ADL, a lower comorbidity index score and a higher baseline frailty index score were associated with the high fatigability and low mobility trajectories.

## Supplementary Information

**Additional file 1.**

## Data Availability

The datasets used during the current study are available from the corresponding author upon reasonable request.

## List of abbreviations

ADL: Activities of Daily Living
BIC: Bayesian Information Criterion
CCI: Charlson Comorbidity Index
DEMMI: De Morton Mobility Index
GBTM: Group-based Trajectory Modeling
GDS: Geriatric Depression Scale
iADL: Instrumental Activities of Daily Living
IQR: Inter Quartile Range
LoS: Length of Stay
OR: Odds Ratio
PFS: Pittsburgh Fatigability Scale
P.O. Box: Post Office Box
Ref: Reference category
SD: Standard Deviation

## References

[1] Henoch I, Sawatzky R, Falk H, Fridh I, Jakobsson Ung E, Sarenmalm EK (2014). Symptom distress profiles in hospitalized patients in sweden: A cross-sectional study. Res Nurs Heal..

[2] van Seben R, Reichardt LA, Aarden JJ, van der Schaaf M, van der Esch M, Engelbert RHH (2019). The Course of Geriatric Syndromes in Acutely Hospitalized Older Adults: The Hospital-ADL Study. J Am Med Dir Assoc..

[3] van Seben R, Reichardt LA, Essink DR, van Munster BC, Bosch JA, Buurman BM (2019). “I Feel Worn Out, as if i Neglected Myself”: Older Patients’ Perspectives on Post-hospital Symptoms after Acute Hospitalization. Gerontologist..

[4] Eldadah BA (2010). Fatigue and Fatigability in Older Adults. PM R..

[5] Simonsick EM, Glynn NW, Jerome GJ, Shardell M, Schrack JA, Ferrucci L (2016). Fatigued, but Not Frail: Perceived Fatigability as a Marker of Impending Decline in Mobility-Intact Older Adults. J Am Geriatr Soc..

[6] Simonsick EM, Schrack JA, Santanasto AJ, Studenski SA, Ferrucci L, Glynn NW (2018). Pittsburgh Fatigability Scale: One-Page Predictor of Mobility Decline in Mobility-Intact Older Adults. J Am Geriatr Soc..

[7] Wanigatunga AA, Simonsick EM, Zipunnikov V, Spira AP, Studenski S, Ferrucci L (2018). Perceived Fatigability and Objective Physical Activity in Mid- to Late-Life. Journals Gerontol - Ser A Biol Sci Med Sci..

[8] Loyd C, Do ADM, Zhang Y, Fowler M, Harper S, Wright NC (2019). Prevalence of Hospital-Associated Disability in Older Adults : A Meta-analysis. J Am Med Dir Assoc..

[9] Chen CC-H, Wang C, Huang G-H (2008). Functional Trajectory 6 Months Posthospitalization. Nurs Res..

[10] Boyd C, Ricks M, Fried LP, Guralnik JM, Xue Q, Bandeen-Roche K (2009). Functional decline and recovery of activities of daily living in hospitalized, disabled older women: the Women’s Health and Aging Study I. J Am Geriatr Soc..

[11] Dasgupta M, Brymer C (2015). Poor functional recovery after delirium is associated with other geriatric syndromes and additional illnesses. Int Psychogeriatrics..

[12] Huang HT, Chang CM, Liu LF, Lin HS, Chen CH (2013). Trajectories and predictors of functional decline of hospitalised older patients. J Clin Nurs..

[13] Brown CJ, Friedkin RJ, Inouye SK (2004). Prevalence and Outcomes of Low Mobility in Hospitalized Older Patients. J Am Geriatr Soc..

[14] Volpato S, Cavalieri M, Sioulis F, Guerra G, Maraldi C, Zuliani G (2011). Predictive Value of the Short Physical Performance Battery Following Hospitalization in Older Patients. Journals Gerontol Ser A Biol Sci Med Sci..

[15] Fried LP, Ferrucci L, Darer J, Williamson JD, Anderson G (2004). Untangling the Concepts of Disability, Frailty, and Comorbidity: Implications for Improved Targeting and Care. Journals Gerontol - Ser A Biol Sci Med Sci..

[16] Xue QL (2011). The Frailty Syndrome: Definition and Natural History. Clin Geriatr Med..

[17] Searle SD, Mitnitski A, Gahbauer EA, Gill TM, Rockwood K (2008). A standard procedure for creating a frailty index. BMC Geriatr..

[18] Laan W, Zuithoff NPA, Drubbel I, Bleijenberg N, Numans ME, de Wit NJ (2014). Validity and reliability of the Katz-15 scale to measure unfavorable health outcomes in community-dwelling older people. J Nutr Health Aging..

[19] Whooley MA, Avins AL, Miranda J, Browner WS (1997). Case-finding instruments for depression: Two questions are as good as many. J Gen Intern Med..

[20] Sheikh, J.I., & Yesavage JA. Geriatric Depression Scale (GDS). Recent evidence and development of a shorter version. In: Brink TL, editor. Gerontology: A Guide to Cliniical Assessment and Intervention. New York: The Haworth Press, Inc.; 1986. p. 165–73.

[21] Pocklington C, Gilbody S, Manea L, McMillan D (2016). The diagnostic accuracy of brief versions of the Geriatric Depression Scale: a systematic review and meta-analysis. Int J Geriatr Psychiatry..

[22] Charlson ME, Pompei P, Ales KL, MacKenzie CR (1987). A new method of classifying prognostic comorbidity in longitudinal studies: development and validation. J Chronic Dis..

[23] Glynn NW, Santanasto AJ, Simonsick EM, Boudreau RM, Beach SR, Schulz R (2015). The Pittsburgh fatigability scale for older adults: Development and validation. J Am Geriatr Soc..

[24] Feenstra M, Smidt N, van Munster BC, Glynn NW, de Rooij SE (2020). Translation and validation of the Dutch Pittsburgh Fatigability Scale for older adults. BMC Geriatr..

[25] de Morton NA, Davidson M, Keating JL (2008). The de Morton Mobility Index (DEMMI): An essential health index for an ageing world. Health Qual Life Outcomes..

[26] Jans MP, Slootweg VC, Boot CR, De Morton NA, Van Der Sluis G, Van Meeteren NL (2011). Reproducibility and validity of the Dutch translation of the de Morton Mobility Index (DEMMI) used by physiotherapists in older patients with knee or Hip osteoarthritis. Arch Phys Med Rehabil..

[27] Nagin D (2005). Group-based modeling of development.

[28] Nagin DS, Odgers CL (2010). Group-based trajectory modeling in clinical research. Annu Rev Clin Psychol..

[29] Theou O, Rockwood MRH, Mitnitski A, Rockwood K (2012). Disability and co-morbidity in relation to frailty: How much do they overlap?. Arch Gerontol Geriatr..

[30] Cooper R, Popham M, Santanasto AJ, Hardy R, Glynn NW, Kuh D (2019). Are BMI and inflammatory markers independently associated with physical fatigability in old age?. Int J Obes..

[31] Jones BL, Nagin DS (2013). A Note on a Stata Plugin for Estimating Group-based Trajectory Models. Sociol Methods Res..

[32] Clegg A, Young J (2011). The frailty syndrome. Clin Med (Northfield Il)..

[33] Hackshaw A (2008). Small studies: Strengths and limitations. Eur Respir J..

