# Supplementary material for: Determinants of trajectories of fatigability and mobility among older medical patients during and after hospitalization; an explorative study
Source: BMC Geriatr. 2022 Jan 3;22:12. doi: 10.1186/s12877-021-02714-9 (PMC8721977; doi:10.1186/s12877-021-02714-9)
Supplement: Supplementary file 1 — Additional file 1. [file 12877_2021_2714_MOESM1_ESM.docx]

ELECTRONIC SUPPLEMENTARY MATERIAL

Contents

[Table S1. Moments and type of assessments. 2](#_Toc70664381)

[Table S2. Item description used for the Frailty Index. 3](#_Toc70664382)

[Table S3. Charlson Comorbidity Index item scores 5](#_Toc70664383)

[Table S4. Reason for admission of all study participants 6](#_Toc70664384)

[Table S5. Group characteristics of patients with missing and complete data 7](#_Toc70664385)

[Table S6. Univariable regression estimates of the the fatigability and mobility trajectories 8](#_Toc70664386)

[Table S7. Univariable regression estimates of the fatigability trajectories. 9](#_Toc70664387)

[Table S8. Univariable regression estimates of the mobility trajectories 10](#_Toc70664388)

[Table S9. Pearson Correlation Coefficients of the independent covariables. 11](#_Toc70664389)

References………………………………………………………………………………………….. 12

| Table S1. Moments and type of assessments including time between the moments of assessments. | | | | | | |
| --- | --- | --- | --- | --- | --- | --- |
| **Study activity** |  | Baseline assessment | Discharge assessment |  | 1^st^ follow-up assessment | 2^nd^ follow-up assessment |
| **Days between assessments, median (IQR)** |  | 3 (2; 4)^#^ | 6 (4; 8) |  | 95 (91; 102) | 93 (86; 105) |
| **Questionnaires** | | | | | | |
| ***Demographics*** | | | | | | |
| Sex |  |  |  |  |  |  |
| Date of birth |  |  |  |  |  |  |
| Education |  |  |  |  |  |  |
| Marrital status |  |  |  |  |  |  |
| Living situation |  |  |  |  |  |  |
| ***Geriatric syndrome*** | | | | | | |
| VMS |  |  |  |  |  |  |
| Number of medication |  |  |  |  |  |  |
| Hearing & vision problems |  |  |  |  |  |  |
| ***Cognition + psychological*** | | | | | | |
| MOCA |  |  |  |  |  |  |
| GDS-2 / 15 |  |  |  |  |  |  |
| CES-D |  |  |  |  |  |  |
| SRH |  |  |  |  |  |  |
| ***Physical functioning*** | | | | | | |
| Katz ADL Index |  |  |  |  |  |  |
| Walk outside |  |  |  |  |  |  |
| Physical exercise |  |  |  |  |  |  |
| EQ-5D |  |  |  |  |  |  |
| NRS pain |  |  |  |  |  |  |
| PFS |  |  |  |  |  |  |
| **Physical test** | | | | | | |
| DEMMI |  |  |  |  |  |  |
| **Other^$^** | | | | | | |
| CCI |  |  |  |  |  |  |
| Length of stay |  |  |  |  |  |  |

# Median number of days between hospital admission and baseline assessment.

$ Retrieved from electronic patient database

Abbreviations: CCI, Charlson Comorbidity Index (1); DEMMI, De Morton Mobility Index (2,3); EQ-5D, EuroQoL 5D (4); GDS, Geriatric Depression Scale (5); Katz ADL Index, 15 items of instrumental and basic activities of daily living (6); MOCA, Montreal Cognitive Assessment (7,8); NRS, numeric Rating Scale; PFS, Pittsburgh Fatigability Scale (9,10); SRH, self-rated health; VMS, Dutch national patient safety program(11).

## Table S2. Item description used for the Frailty Index (12).

| **Variable** | **Scoring** |
| --- | --- |
| **ADL / iADL domain** | |
| 1. Help bathing | Yes = 1  No = 0 |
| 1. Help dressing |  |
| 1. Help grooming |  |
| 1. Help using toilet |  |
| 1. Difficulty getting out chair |  |
| 1. Help getting out chair |  |
| 1. Help walking |  |
| 1. Help eating |  |
| 1. Help using phone |  |
| 1. Help transport |  |
| 1. Help shopping |  |
| 1. Help meal preparations |  |
| 1. Help housework |  |
| 1. Help taking medications |  |
| 1. Help finances |  |
| **Geriatric syndrome** | |
| 1. Urinary incontinence | Yes = 1  No = 0 |
| 1. Visual impairment |  |
| 1. Hearing impairment |  |
| 1. Fall in past month |  |
| 1. Unintentional weight loss |  |
| 1. Concurrent use of ≥5 medications |  |
| **Psychosocial domain** | |
| 1. Feel everything is an effort | Yes = 1 No = 0 |
| 1. Difficulty get going |  |
| 1. Self-rated health | Poor = 1 Fair = 0.75  Good = 0.5 Very Good = 0.25 Excellent = 0 |
| 1. Did you feel downhearted and blue? 2. Have you dropped many of your interests and activities? | Both yes = 1  One yes = 0.5  Both no = 0 |
| **Symptoms** | |
| 1. Pain / discomfort | No pain / discomfort = 0 Moderate = 0.5 Extreme = 1 |
| 1. Mood | Not anxious / depressed = 0  Moderately = 0.5 Extreme = 1 |
| 1. Loss of appetite^*^ | Yes = 1 No = 0 |
| **Physical domain** | |
| 1. Housing situation | Independent / conventional = 0 Transitional = 0.5  Institutionalized = 1 |
| 1. Living situation | Living alone = 1  Cohabiting/married = 0 |
| 1. Walk outside for 5 minutes | Impossible = 1 Only with help of somebody else = 0.75 Much effort = 0.5 Some effort = 0.25 No effort = 0 |
| 1. Physical exercise for at least 30 minutes | Never = 1 Monthly = 0.75  Weekly = 0.5  3 days/week = 0.25 Daily = 0 |
| **Cognitive domain** | |
| 1. MOCA^†^ | Total score ≥26 = 0  Total score ≤25 = 1 |

Notes: All baseline assessments referred to the situation two weeks before hospital admission unless indicated otherwise. (i)ADL, (instrumental) Activities of Daily Living; MOCA, Montreal Cognitive Assessment (7,8).

* Baseline assessment referred to a month before hospital admission.

^†^ The MOCA was assessed within the first four days of hospital admission.

## Table S3. Charlson Comorbidity Index (1) item scores within the joint trajectories of fatigability and mobility.

|  |  |  |  | **Trajectory group^a^** | | |
| --- | --- | --- | --- | --- | --- | --- |
|  | | **Total** |  | **Low fatigability high mobility** | **Improving fatigability  high mobility** | **High fatigability**  **low mobility** |
| **N (%)** | | 44 (100) |  | 5 (11) | 23 (52) | 16 (36) |
| **Comorbidity Index, median (IQR)** | | 2 (1; 3) |  | 3 (2; 5) | 2 (1; 3) | 2 (1; 3) |
|  | **Acute myocardial infarction^*^** | 4 |  | 0 | 2 | 2 |
|  | **Congestive heart failure^*^** | 11 |  | 0 | 6 | 5 |
|  | **Peripheral vascular disease^*^** | 3 |  | 1 | 1 | 1 |
|  | **Cerebrovascular Accident^*^** | 4 |  | 0 | 4 | 0 |
|  | **Dementia^*^** | - |  | - | - | - |
|  | **Chronic lung disease^*^** | 18 |  | 0 | 9 | 8 |
|  | **Rheumatic disease^*^** | 4 |  | 1 | 2 | 1 |
|  | **Peptic ulcer^*^** | 2 |  | 1 | 0 | 1 |
|  | **Mild liver disease^*^** | 0 |  |  |  |  |
|  | **Mild to moderate diabetes^*^** | 4 |  | 0 | 2 | 2 |
|  | **Diabetes with chronic complications^†^** | 1 |  | 0 | 0 | 1 |
|  | **Hemi- / paraplegia^†^** | - |  | - | - | - |
|  | **Kidney disease^†^** | 1 |  | 1 | 0 | 0 |
|  | **Malignant tumors^†^** | 6 |  | 1 | 4 | 1 |
|  | **Leukemia^†^** | 1 |  | 0 | 1 | 0 |
|  | **Lymphoma^†^** | 2 |  | 0 | 0 | 2 |
|  | **Moderate to serious liver disease^**^** | 1 |  | 0 | 1 | 0 |
|  | **Solid, metastatic tumor^‡^** | 3 |  | 1 | 1 | 1 |
|  | **AIDS^‡^** | - |  | - | - | - |
| ***Missing data for CCI*** | | *2* |  | *1* | *0* | *1* |

^*^ counts for 1 point to the Charlson Cormordity Index total score.

^†^ counts for 2 points to the Charlson Cormordity Index total score.

^**^ counts for 3 points to the Charlson Cormordity Index total score.

^‡^ counts for 6 points to the Charlson Cormordity Index total score.

| Table S4*.* Reason for admission of all study participants |
| --- |

|  |  |  |  | **Trajectory group^a^** | | |
| --- | --- | --- | --- | --- | --- | --- |
|  | | **Total** |  | **Low fatigability high mobility** | **Improving fatigability  high mobility** | **High fatigability**  **low mobility** |
| **N (%)** | | 44 (100) |  | 5 (11) | 23 (52) | 16 (36) |
| **Reason for admission** | |  |  |  |  |  |
|  | **Diagnostic^*^** | 5 (11) |  | 1 (20) | 4 (80) | 0 (0) |
|  | **Therapeutic^†^** | 38 (86) |  | 4 (11) | 19 (50) | 15 (39) |
|  | ***Curative*** | 36 *(95)* |  | 4 *(100)* | 17 *(89)* | 15 *(100)* |
|  | ***Palliative*** | 2 *(5)* |  | 0 *(0)* | 2 *(11)* | 0 *(0)* |
|  | ***Missing*** | *1 (2)* |  |  |  |  |

^*^ Patients whose reason for admission was for diagnostic evaluation

^†^ Patients were admitted to the hospital after a diagnosis was established.

## Table S5. Group characteristics of patients with missing and complete data for the main outcomes.

| **Baseline characteristic** | **Not missing DEMMI data** | | **Missing DEMMI data** | | **Not missing PFS data** | | **Missing PFS data** |
| --- | --- | --- | --- | --- | --- | --- | --- |
| **N (%)** | 39 (89) | | 5 (11) | | 35 (80) | | 9 (20) |
| **Demographic characteristics** |  | |  | |  | |  |
| Age in years, mean (SD) | 77 (5.5) | | 74 (7.5) | | 76.7 (5.5) | | 77.2 (5.9) |
| Female sex | 20 (51) | | 2 (40) | | 20 (57) | | 2 (22) |
| <12 years of education | 16 (42) | | 2 (40) | | 14 (41) | | 4 (44) |
| Living alone | 12 (31) | | 1 (20) | | 12 (34) | | 1 (11) |
| **Clinical characteristics** |  | |  | |  | |  |
| Frailty^†^, mean (SD) | 0.25 (0.15) | 0.31 (0.15) | | 0.26 (0.14) | | 0.24 (0.15) | |
| ≥1 disabilities | 26 (67) | | 3 (75) | | 24 (69) | | 5 (62) |
| ≥1 disabilities basic ADL | 15 (38) | | 3 (60) | | 15 (43) | | 3 (33) |
| ≥1 disabilities iADL | 23 (59) | | 3 (75) | | 22 (63) | | 4 (50) |
| Depressive symptoms^‡^ | 7 (18) | | 1 (20) | | 7 (20) | | 1 (11) |
| Comorbidity^§^, mean (SD) | 2.4 (2.0) | | 1.8 (0.8) | | 2.5 (2.0) | | 1.2 (1.3) |
| LoS in days, mean (SD) | 6.2 (4.2) | | 6.6 (4.3) | | 6 (4.1) | | 7.2 (4.7) |
| Fatigability^\|^, mean (SD) | 30.7 (9.0) | | 27.7 (8.5) | | - | | - |
| Mobility^¶^, mean (SD) | - | | - | | 66 (22) | | 59 (28) |

Results are presented as n (%) unless indicated otherwise.

†. Frailty was assessed by the Frailty Index (12).

‡. Depressive symptoms were positive when the Geriatric Depression Score was ≥5 (5).

§. Comorbidity was assessed by the Charlson Comorbidity Index (1).

|. Fatigability was assessed by the physical subscale of the PFS (9,10).

¶ Mobility was assessed by the DEMMI (2,3).

Abbreviations: ADL, activities of daily living; DEMMI, De Morton Mobility Index; IQR, interquartile range; LoS, length of stay; PFS, Pittsburgh Fatigability Scale.

## Table S6. Univariable regression estimates (odds ratio’s and 95% confidence intervals) of the associations between the dual fatigability and mobility trajectories^#^ and demographic and clinical characteristics using the improving fatigability high mobility trajectory group as the reference group.

| **Characteristic** | **Low fatigability  high mobility** | **Improving fatigability high mobility** | **High fatigability low mobility** |
| --- | --- | --- | --- |
| Age, per year | 1.06 (0.89; 1.27) | Ref. | 1.09 (0.97; 1.23) |
| Sex |  |  |  |
| Female | 0.87 (0.12; 6.21) | Ref. | 2.17 (0.59; 7.99) |
| Male |  | Ref. |  |
| Education |  |  |  |
| <12 years of education | 1.75 (0.16; 18.62) | Ref. | 0.22 (0.05; 0.88)* |
| ≥12 years of education |  | Ref. |  |
| Living situation |  |  |  |
| Living alone | 2.40 (0.31; 18.55) | Ref. | 2.16 (0.52; 8.90) |
| Living together |  | Ref. |  |
| Functional status |  |  |  |
| No baseline disabilities |  | Ref. |  |
| ≥1 baseline disabilities | 0.17 (0.02; 1.82) | Ref. | 10.38 (1.16; 93.29)* |
| Frailty^†^, per 0.01 point | 0.93 (0.83; 1.04) | Ref. | 1.17 (1.06; 1.29)* |
| Depressive symptoms |  |  |  |
| No depressive symptoms |  | Ref. |  |
| Depressive symptoms | 2.63 (0.19; 36.34) | Ref. | 4.77 (0.79; 28.72) |
| Comorbidity^$^, per point | 1.35 (0.84; 2.15) | Ref. | 1.04 (0.73; 1.49) |
| Length of stay, per day | 0.90 (0.63; 1.28) | Ref. | 1.12 (0.96; 1.31) |

^#^ Trajectory groups were estimated by jointly modeling fatigability and mobility over four waves from hospital admission to six months post discharge. The improving fatigability high mobility trajectory was used as the reference category.

^†^ Frailty was assessed using the Frailty Index (12).

^$^ Comorbidity was assessed using the Charlson Comorbdity Index (1).

* p: 0.05

## Table S7. Univariable regression estimates (odds ratio’s and 95% confidence intervals) of the associations between the fatigability trajectories and demographic and clinical characteristics.

| **Characteristic** | **Low fatigability** | **Improving fatigability** | **Stable high fatigability** |
| --- | --- | --- | --- |
| Age. per year | Ref. | 0.96 (0.80; 1.15) | 1.00 (0.84; 1.19) |
| Sex | Ref. |  |  |
| Female | Ref. | 1.14 (0.15; 8.59) | 0.90 (0.05; 2.68) |
| Male | Ref. | Ref. | Ref. |
| Education | Ref. |  |  |
| <12 years of education | Ref. | 0.70 (0.06; 7.85) | 0.82 (0.01; 1.58) |
| ≥12 years of education | Ref. | Ref. | Ref. |
| Living situation | Ref. |  |  |
| Living alone | Ref. | 0.88 (0.03; 2.47) | 1.00 (0.14; 7.39) |
| Living together | Ref. | Ref. | Ref. |
| Functional status | Ref. |  |  |
| No baseline disabilities | Ref. | Ref. | Ref. |
| ≥1 baseline disabilities | Ref. | 5.50 (0.51; 59.01) | 34.00 (2.44; 474.55)** |
| Frailty^†^, per 0.01 point | Ref. | 1.00 (1.00; 1.00) | 1.21 (1.06; 1.39)** |
| Depressive symptoms | Ref. |  |  |
| No depressive symptoms | Ref. | Ref. | Ref. |
| Depressive symptoms | Ref. | 0.47 (0.03; 6.57) | 1.33 (0.12; 14.90) |
| Comorbidity^#^, per point | Ref. | 0.79 (0.49; 1.25) | 0.72 (0.44; 1.18) |
| Length of stay, per day | Ref. | 1.13 (0.80; 1.61) | 1.20 (0.85; 1.71) |
| Baseline mobility^$^, per point | Ref. | 1.01 (0.95; 1.07) | 0.93 (0.87; 0.99)* |

^†^ Frailty was assessed using the Frailty Index (12).

^#^ Comorbidity was assessed using the Charlson Comorbidity Index (1) .
^$^ Mobility was assessed using the De Morton Mobility Index (2,3).

* p≤ 0.05, ** p≤0.01

## Table S8. Univariable regression estimates (odds ratio’s and 95% confidence intervals) of the associations between the mobility trajectories and demographic and clinical characteristics.

| **Characteristic** | **High mobility** | **Low mobility** |
| --- | --- | --- |
| Age, per year | Ref. | 1.17 (1.03; 1.32)* |
| Sex | Ref. |  |
| Female | Ref. | 0.54 (0.15; 1.92) |
| Male | Ref. | Ref. |
| Education | Ref. |  |
| <12 years of education | Ref. | 0.87 (0.07; 0.96)* |
| ≥12 years of education | Ref. | Ref. |
| Living situation | Ref. |  |
| Living alone | Ref. | 2.10 (0.55; 7.99) |
| Living together | Ref. | Ref. |
| Functional status | Ref. |  |
| No baseline disabilities | Ref. | Ref. |
| ≥1 baseline disabilities | Ref. | 12.13 (1.40; 105.25)* |
| Frailty^†^, per 0.01 point | Ref. | 1.13 (1.05; 1.22)** |
| Depressive symptoms | Ref. |  |
| No depressive symptoms | Ref. | Ref. |
| Depressive symptoms | Ref. | 4.33 (0.87; 21.60) |
| Comorbidity^#^, per point | Ref. | 1.08 (0.78; 1.49) |
| Length of stay, per day | Ref. | 1.20 (1.01; 1.41)* |
| Baseline fatigability^$^, per point | Ref. | 1.23 (106; 1.44)** |

^†^ Frailty was assessed using the Frailty Index (12).

^#^ Comorbidity was assessed using the Charlson Comorbidity Index (1).
^$^ Fatigability was assessed using the Pittsburgh Fatigability Scale (9,10).

* p≤ 0.05, ** p≤0.01

## Table S9. Pearson Correlation Coefficients of the independent covariables.

| **Variables** | | 1 | 2 | 3 | 4 | 5 | 6 | 7 | 8 | 9 |
| --- | --- | --- | --- | --- | --- | --- | --- | --- | --- | --- |
| 1 | Age | 1.00 |  |  |  |  |  |  |  |  |
| 2 | Sex | 0.03 | 1.00 |  |  |  |  |  |  |  |
| 3 | Education | -0.31 | 0.37 | 1.00 |  |  |  |  |  |  |
| 4 | Living situation | -0.27 | 0.40 | 0.35 | 1.00 |  |  |  |  |  |
| 5 | Frailty | 0.07 | -0.15 | -0.37 | -0.24 | 1.00 |  |  |  |  |
| 6 | ADL disabilities^*^ | -0.07 | -0.20 | -0.24 | 0.01 | 0.56 | 1.00 |  |  |  |
| 7 | Depressive symptoms^*^ | 0.19 | -0.18 | -0.15 | 0.06 | 0.31 | 0.31 | 1.00 |  |  |
| 8 | Comorbidity | -0.07 | 0.02 | 0.24 | -0.12 | 0.33 | 0.22 | 0.30 | 1.00 |  |
| 9 | LoS | 0.12 | 0.16 | -0.11 | 0.01 | 0.38 | 0.21 | 0.23 | 0.32 | 1.00 |

Abbreviations: ADL, Activities of Daily Living; Los, Length of hospital Stay.

^*^ Scoring as used in the analyses.

## References

1. Charlson ME, Pompei P, Ales KL, MacKenzie CR. A new method of classifying prognostic comorbidity in longitudinal studies: Development and validation. J Chronic Dis. 1987;40(5):373–83.

2. de Morton NA, Davidson M, Keating JL. The de Morton Mobility Index (DEMMI): An essential health index for an ageing world. Health Qual Life Outcomes. 2008;6(1):63.

3. Jans MP, Slootweg VC, Boot CR, De Morton NA, Van Der Sluis G, Van Meeteren NL. Reproducibility and validity of the Dutch translation of the de Morton Mobility Index (DEMMI) used by physiotherapists in older patients with knee or Hip osteoarthritis. Arch Phys Med Rehabil . 2011;92(11):1892–9.

4. The EuroQol Group. EuroQol-a new facility for the measument of health related Quality of Life. Health Policy (New York). 1990;16(3):199–208.

5. Sheikh, J.I., & Yesavage JA. Geriatric Depression Scale (GDS). Recent evidence and development of a shorter version. In: Brink TL, editor. Gerontology: A Guide to Cliniical Assessment and Intervention. New York: NY: The Haworth Press, Inc.; 1986. p. 165–73.

6. Laan W, Zuithoff NPA, Drubbel I, Bleijenberg N, Numans ME, de Wit NJ, et al. Validity and reliability of the Katz-15 scale to measure unfavorable health outcomes in community-dwelling older people. J Nutr Health Aging. 2014;18(9):848–54.

7. Nasreddine ZS, Phillips NA, Bedirian V, Charbonneau S, Whitehead V, Collin I, et al. The Montreal Cognitive Assessment, MoCA: A Brief Screening. J Am Geriatr Soc. 2005;695–9.

8. Thissen AJAM, Van Bergen F, De Jonghe JFM, Kessels RPC, Dautzenberg PLJ. Bruikbaarheid en validiteit van de Nederlandse versie van de Montreal Cognitive Assessment (MoCA-D) bij het diagnosticeren van Mild Cognitive Impairment [Applicability and validity of the Dutch version of the Montreal Cognitive Assessment (MoCA-D) in diagnosing MCI. Tijdschr Gerontol Geriatr. 2010;41(6):231–40.

9. Glynn NW, Santanasto AJ, Simonsick EM, Boudreau RM, Beach SR, Schulz R, et al. The Pittsburgh fatigability scale for older adults: Development and validation. J Am Geriatr Soc. 2015;63(1):130–5.

10. Feenstra M, Smidt N, van Munster BC, Glynn NW, de Rooij SE. Translation and validation of the Dutch Pittsburgh Fatigability Scale for older adults. BMC Geriatr. 2020;20(1):234.

11. de Rooij SEJA, Emmelot-Vonk MH, Evers A, Knijnenburg CMR, Kok RM, Nijs K, et al. Kwetsbare ouderen. Den Haag; 2009.

12. Searle SD, Mitnitski A, Gahbauer EA, Gill TM, Rockwood K. A standard procedure for creating a frailty index. BMC Geriatr. 2008;8(1):24.
